# Wildlife as Source of Zoonotic Infections

**DOI:** 10.3201/eid1012.040707

**Published:** 2004-12

**Authors:** Hilde Kruse, Anne-Mette Kirkemo, Kjell Handeland

**Affiliations:** * National Veterinary Institute, Oslo, Norway

**Keywords:** Zoonoses, wildlife reservoir, public health, transmission modes, epidemiology, prevention, control, perspective

## Abstract

Zoonoses with a wildlife reservoir represent a major public health problem, affecting all continents. Hundreds of pathogens and many different transmission modes are involved, and many factors influence the epidemiology of the various zoonoses. The importance and recognition of wildlife as a reservoir of zoonoses are increasing. Cost-effective prevention and control of these zoonoses necessitate an interdisciplinary and holistic approach and international cooperation. Surveillance, laboratory capability, research, training and education, and communication are key elements.

## Abstract 2

Zoonoses with a wildlife reservoir represent a major public health problem, affecting all continents.

**Figure 1:**
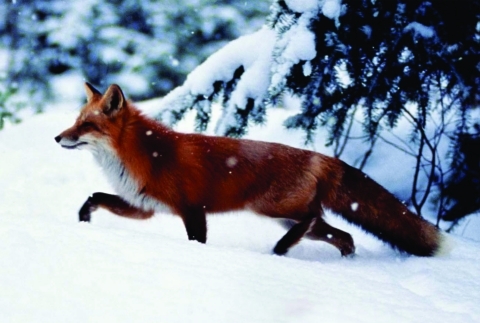
Foxes may be a reservoir of zoonotic agents such as rabies virus and the parasite *Echinococcus multilocularis*.

**Figure 2:**
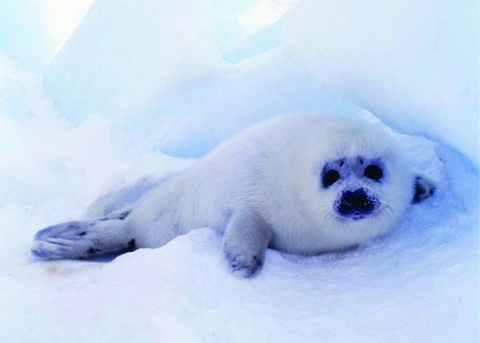
The pathologic role of marine *Brucella* spp. in animals, such as pinnipedes, remains unclear, as does their zoonotic potential.

Throughout history, wildlife has been an important source of infectious diseases transmissible to humans. Today, zoonoses with a wildlife reservoir constitute a major public health problem, affecting all continents. The importance of such zoonoses is increasingly recognized, and the need for more attention in this area is being addressed.

Wildlife is normally defined as free-roaming animals (mammals, birds, fish, reptiles, and amphibians), whereas a zoonosis is an infectious disease transmittable between animals and humans. The total number of zoonoses is unknown, but according to Taylor et al. (*1*), who in 2001 catalogued 1,415 known human pathogens, 62% were of zoonotic origin. With time, more and more human pathogens are found to be of animal origin. Moreover, most emerging infectious diseases in humans are zoonoses. Wild animals seem to be involved in the epidemiology of most zoonoses and serve as major reservoirs for transmission of zoonotic agents to domestic animals and humans.

Zoonoses with a wildlife reservoir are typically caused by various bacteria, viruses, and parasites, whereas fungi are of negligible importance. Regarding prion diseases, chronic wasting disease occurs among deer in North America. This prion disease is thus far not known to be zoonotic. However, hunters and consumers are advised to take precautions (*2**,**3*).

## Historical Aspects

Zoonoses have affected human health throughout times, and wildlife has always played a role. For example, bubonic plague, a bacterial disease for which rats and fleas play a central role in transmission, has caused substantial illness and death around the world since ancient times (*4*). A possible epidemic of bubonic plague was described in the Old Testament, in the First Book of Samuel. The so-called Black Death emerged in the 14th century and caused vast losses throughout Asia, Africa, and Europe. The epidemic, which originated in the Far East, killed approximately one third of Europe's population. However, bubonic plague still occurs in Asia, Africa, and the Americas, and the World Health Organization annually reports 1,000–3,000 cases. In the western United States, acquisition of plague in humans is linked to companion animals infested with *Yersinia pestis*–carrying fleas in areas of endemic sylvatic disease (*5*).

Rabies was described in Mesopotamia, in hunting dogs, as early as 2,300 BC. Recognizable descriptions of rabies can also be traced back to early Chinese, Egyptian, Greek, and Roman records (*6*). In Europe in the medieval age, rabies occurred in both domestic animals and wildlife. Rabid foxes, wolves, badgers, and bears have been described in the literature as well as in figurative art.

Ancient accounts and modern hypotheses suggest that Alexander the Great, who died in Babylon in 323 BC, died of encephalitis caused by West Nile virus (*7*), a virus that has a wild bird reservoir. Marr and Calisher reported that as Alexander entered Babylon, a flock of ravens exhibiting unusual behavior died at his feet (*7*). In 1999, West Nile virus was introduced into the United States, where it caused the ongoing epizootic in birds with a spillover of infections to humans and equines.

## Transmission Modes

Zoonoses with a wildlife reservoir represent a large spectrum of transmission modes. Several zoonotic agents can be directly transmitted from wildlife to humans, e.g., *Francisella tularensis*, the causative agent of tularemia, can be transmitted by skin contact with an infected, diseased, or dead hare or rodent. By contrast, rabies virus is transmitted by bite (saliva) from a rabid animal. Hantaviruses are spread from rodents to humans by aerosols in dust from rodent excreta. Zoonotic agents can also be spread from wildlife to humans indirectly by contaminated food and water, for example *Salmonella* spp. and *Leptospira* spp.

Many zoonoses with a wildlife origin are spread through insect vectors. For example, mosquitoes are well-known vectors of several wildlife zoonoses, such as Rift Valley fever, equine encephalitis, and Japanese encephalitis. *Y. pestis* can be spread by fleas, *Bacillus anthracis* spores by flies, and *Leishmania* by sand-flies, whereas ticks are essential in the spread of *Borrelia burgdorferi* and *Ehrlichia/Anaplasma*.

A good example of a zoonotic agent with many different transmission modes is *F. tularensis*. Rodents and hares constitute the main sources of infections, and hunters are at particular risk of acquiring the disease. The transmission mode also affects the clinical manifestation in humans. The agent can be transmitted by direct contact through the handling of an infected carcass and through tick or mosquito bites, which cause initial skin symptoms such as ulcers. Infection may also occur after eating insufficiently cooked meat from an infected animal or drinking contaminated water, causing symptoms from the digestive tract, and by inhalation of contaminated dust, causing a pneumonialike illness.

*Salmonella* spp. can also be spread from wildlife to humans in different ways. Reptile-associated salmonellosis is a well-described phenomenon, especially among children. The increasing popularity of keeping reptiles and other exotic animals as pets presents a public health problem, as such animals are commonly carriers of *Salmonella* spp. and thereby can infect humans directly or indirectly. In Norway, special types of *Salmonella enterica* subsp. *enterica* serovar Typhimurium (*S*. Typhimurium) occur endemically in hedgehogs and wild passerine birds, causing sporadic cases and small outbreaks in humans. In 1987, a nationwide outbreak of *S*. Typhimurium infections was traced to chocolate bars that had been contaminated by wild birds in the factory. In 1999, a waterborne outbreak of *S*. Typhimurium infections was linked to a dead seagull that had contaminated a reservoir water source from which the water was used untreated (*8**–**10*).

*B. anthracis*, the etiologic agent of anthrax, primarily a disease of herbivores, can also be transmitted from wildlife to humans by various modes. The spores formed by the bacteria are very resistant and have been found to remain dormant and viable in nature for >100 years (*11*). Anthrax is spread by food and water contamination or by the spread of spores by flies, vultures, and other scavengers. Humans can be infected by eating meat from infected carcasses or drinking contaminated water, through the skin by contact with infected material or by insect bites, and through the lungs by inhaling spores. Although livestock anthrax is declining in many parts of the world, the disease remains enzootic in many national parks, for example, in southern Africa and North America. Anthrax in wildlife represents a persistent risk for surrounding livestock and public health (*12*).

## Factors Influencing the Epidemiology of Zoonoses with a Wildlife Reservoir

The ecologic changes influencing the epidemiology of zoonoses with a wildlife reservoir can be of natural or anthropogenic origin. These include, but are not limited to, human population expansion and encroachment, reforestation and other habitat changes, pollution, and climatic changes.

The spirochete *Borrelia burgdorferi*, which causes Lyme borreliosis, has its main reservoir among small rodents and uses various *Ixodes* species as vectors (*13*). Lyme borreliosis was first recognized in Lyme, Connecticut, in 1975, and since then, an increasing number of cases have been reported in North America, Europe, and Asia. The increasing incidence of Lyme borreliosis in the northeastern United States in recent years can be explained by reforestation that has favored transmission of the disease through increased populations of white-tailed deer and deer mice and thereby abundance of the tick vector, *Ixodes scapularis*.

Wild rodents also constitute a reservoir of hantaviruses (*14*). The viruses are shed in urine, droppings, and saliva, and humans are mainly infected aerogenically by inhaling aerosols containing the virus. Precipitation, habitat structure, and food availability are critical environmental factors that affect rodent population dynamics as well as viral transmission between animals and subsequently the incidence of human infection. The deer mouse is a reservoir host for Sin Nombre hantavirus, which causes hantavirus pulmonary syndrome in the southwestern United States. Because of climatic changes with increased rainfall in recent years, host abundance, and thereby spread of the pathogen, has increased, with subsequent transmission to humans.

The movement of pathogens, vectors, and animal hosts is another factor influencing the epidemiology of zoonoses with a wildlife reservoir. Such movement can, for example, occur through human travel and trade, by natural movement of wild animals including migratory birds, and by anthropogenic movement of animals. For instance, infectious agents harbored within insects, animals, or humans can travel halfway around the globe in <24 hours in airplanes. Thus, infectious agents can be transported to the farthest land in less time than it takes most diseases to incubate. The appearance of West Nile virus infection in New York in 1999, and the subsequent spread within the United States, is an example of introduction and establishment of a pathogen that apparently originated in the Middle East (*15*).

Movement of infected wild and domestic animals is an important factor in the appearance of rabies in new locations. Rabies virus, which is widely distributed and affects various animals, especially canids, was introduced into North America by infected dogs in the early 18th century, with subsequent spillover to a variety of wild terrestrial mammals. Rabies became established in raccoons in the mid-Atlantic states in the late 1970s when raccoons were translocated from the southeastern United States, where rabies was endemic in this species (*16*). Finland experienced an outbreak of rabies linked to raccoon dogs in 1988. The raccoon dog had spread to Finland after this species was released in western Russia for fur trade. Rabies most probably arrived in Finland by wolves migrating from Russia during wintertime along the ice-packed coast (*17*). In the Arctic, the ice links the continents together. The movement of the arctic fox from the archipelago of Spitzbergen to Novaja Zemlja in Siberia and from Canada to Greenland has been described, indicating another way that rabies can be spread to new areas (*18**,**19*).

Bovine tuberculosis caused by *Mycobacterium bovis* is another zoonosis in which both natural and anthropogenic movement of animals has influenced the epidemiology. This zoonosis is emerging in wildlife in many parts of the world, and wildlife can represent a source of infection for domestic animals and humans. Bovine tuberculosis was probably introduced into Africa with imported cattle during the colonial era and thereafter spread to and became endemic in wildlife (*20*). In Ireland and Great Britain, badgers maintain the infection, whereas the brushtail possum constitutes a main wildlife reservoir in New Zealand. In parts of Michigan, bovine tuberculosis is endemic among white-tailed deer, whereas in Europe, both wild boars and various deer species can be a reservoir of the pathogen. The natural movement of these reservoir animals increases the spread of the disease to domestic animals and thereby its public health impact (*21*).

The epidemiology of multilocular echinococcosis, caused by the small tapeworm *Echinococcus multilocularis*, has also been influenced by the translocation of animals. The main hosts are canids, especially foxes; the intermediate hosts are small rodents. Humans can become accidental intermediate hosts, by ingesting eggs. Multilocular echinococcosis occurs in large parts of the Northern Hemisphere. In 1999, *E. multilocularis* was detected for the first time in Norway, in the archipelago of Spitzbergen (*10**,**22*). The parasite most probably spread from Russia, by natural movement of the main host, the Arctic fox. Establishment of the parasite was possible because the intermediate host, the sibling vole, had previously been translocated to Spitzbergen, most likely through imported animal feed (*23*). In Copenhagen, Denmark, in 2000, *E. multilocularis* was detected in a traffic-killed red fox. The theory is that the fox had traveled by train from central Europe, where the disease is endemic (H.C. Wegener, pers. comm.).

During the summer of 2003, an outbreak of monkeypox occurred in the United States with 37 confirmed human cases (*24*). Monkeypox is a rare zoonosis caused by a poxvirus that typically occurs in Africa. It was first found in monkeys in 1958 and later on in other animals, especially rodents. The African squirrel is probably the natural host. Transmission to humans occurs by contact with infected animals or body fluids. The cases in the United States, the first outside Africa, were associated with contact with infected prairie dogs. The outbreak was epidemiologically linked to imported African rodents from Ghana. Most likely, infected imported rodents have transmitted the virus to prairie dogs in United States. This transmission illustrates how non-native animal species can create serious public health problems when they introduce a disease to native animal and human populations. Thus, the transportation, sale, or distribution of animals, or the release of animals into the environment, can represent a risk for spread of zoonoses.

Microbial changes or adaptation also influence the epidemiology of zoonoses with a wildlife reservoir. These changes include mutations, such as genetic drift in viruses; activation and silencing of genes; genetic recombinations, such as genetic shift in viruses; and conjugation, transformation, and transduction in bacteria. Natural selection and evolution also play a role. Transmission of adaptive or genetically changed microorganisms from wildlife to humans, either directly or indirectly through domestic animals, may occur in many ways. In this respect an international wildlife trade, often illegal, in which wild animals end up in live-animal markets, restaurants, and farms, is important because such practices increase the proximity between wildlife, domestic animals, and humans (*25*).

Severe acute respiratory syndrome (SARS) is a current example of likely microbial adaptation. This viral respiratory illness, caused by SARS-associated coronavirus, is believed to have emerged in Guangdong, China, in November 2002. SARS was first reported in Asia in February 2003, and over the next few months, the illness spread to a global epidemic before it was contained. According to the World Health Organization, 8,098 cases, including 774 fatalities, have occurred during this epidemic. The virus has an unknown reservoir, but wildlife is a likely source of infection. Natural infection has been demonstrated in palm civet cats in markets and also in raccoon dogs, rats, and other animals indigenous to the area where SARS likely originated (*26*).

Genetic changes typically influence the epidemiology of influenza A. Natural infections with influenza A viruses have been reported in a variety of animal species, including birds, humans, pigs, horses, and sea mammals, and its main reservoir seems to be wild waterfowl, especially ducks. Influenza A virus has two main surface antigens; hemagglutinin with 15 subtypes and neuraminidase with 9 subtypes. All these subtypes, in most combinations, have been isolated from birds, whereas few combinations have been found in mammals. In the 20th century, the sudden emergence of antigenically different strains transmissible in humans, termed antigenic shift, has occurred on four occasions, each time resulting in a pandemic. "New" pandemic strains most certainly emerged after reassortment of genes of viruses of avian and human origin in a permissive host (*27*). The H5N1 strain of a highly pathogenic avian influenza that caused a severe outbreak in poultry in Southeast Asia in 2004 (*28*) demonstrated its capacity to infect humans; 39 cases, 28 of them fatal, were officially reported (*29*). For the human population as a whole, the main danger appears to be simultaneous infection with an avian and a human influenza virus. Reassortment could then occur either in humans or in pigs with the potential emergence of a virus fully capable of spread among humans but with antigenic characteristics for which the human population was immunologically naïve.

Enhanced recognition can also result in an apparent change in the epidemiology of a zoonosis, for example, the recognition of an agent that has been present for a long time but was previously undetected because of lack of diagnostic tools. Improved methods for molecular characterization have helped describe a larger repertoire of zoonotic agents.

Recognition and emergence of human tickborne ehrlichiosis are recent and continuing events, beginning with human monocytic ehrlichiosis and human granulocytic ehrlichiosis, reported first in the United States in 1987 and 1994, respectively. The causative agents, *Ehrlichia chaffeensis* and *Anaplasma phagocytophilum*, are intracellular bacteria that are maintained in zoonotic cycles involving persistently infected deer and rodents (*30*).

From 1994 to 2004, three zoonotic paramyxoviruses with a wildlife reservoir have emerged. The Hendra, Menangle, and Nipah viruses all have a fruit bat reservoir (*31*). Humans are infected by close contact with infected pigs or horses. Hendra virus infection was described in Australia in 1994, where it caused acute, fatal respiratory disease in horses and humans. Menangle virus was also described in Australia, in 1996, where it caused reproductive disorders in pigs and an influenzalike disease in humans. Nipah virus was detected in 1998, in Malaysia, when it caused severe disease with respiratory and neurologic symptoms among pigs and encephalitis with a 40% death rate in humans in close contact with pigs.

Since 1994, when the isolation of *Brucella* spp. from marine mammals was reported for the first time, such infections have been detected in a wide range of marine mammal species and populations. The pathologic role of marine *Brucella* spp. in animals remains unclear, as does their zoonotic potential. In 2003, two human cases of community-acquired granulomatous central nervous system infections caused by marine *Brucella* spp. were reported (*32*).

Human behavior and demographic factors can also influence the epidemiology of zoonoses with a wildlife reservoir. Hiking, camping, and hunting are activities that may represent risk factors for acquiring certain zoonoses with a wildlife reservoir, e.g., tickborne zoonoses and tularemia. Eating habits can also play a role. For example, eating meat from exotic animals such as bear increases the risk of acquiring trichinellosis (*33*). AIDS represents a disease in which demographic factors and human behavior have contributed to its development into a global public health problem. The origin of HIV, the virus causing AIDS, is still a matter of controversy, but HIV likely spread to humans from nonhuman primates in West Africa (*34*).

## Prevention and Control

Although prevention and control strategies for the various zoonoses associated with wildlife share many common aspects, specific strategies are also needed to address the etiology and epidemiology of the disease, characteristics of the pathogen involved, ecologic factors, and the population at risk. As wildlife is an essential component in the epidemiology of many, if not most, zoonoses, wildlife should be taken into account in the risk analysis framework. Consequently, cost-effective prevention and control of zoonoses in humans, including risk communication, necessitate an interdisciplinary and holistic approach that acknowledges the importance of wildlife as a reservoir.

To increase the capability of recognizing zoonoses with a wildlife reservoir, better national surveillance systems for humans and animals are needed, as well as better international integration and sharing of information from such systems. Which diseases should be reportable also needs to be evaluated on a continuous basis. Improved reporting systems and screening programs for human infections, including the application of syndromic surveillance, are warranted to detect new and emerging zoonoses. Efficient surveillance is dependent upon a laboratory system that is capable of identifying and characterizing the pathogens in question. More research is needed to better understand the epidemiology and pathogenesis of various zoonoses, to improve diagnostic methods, and to develop cost-effective vaccines and drugs. Training and education are prerequisites to enable the personnel involved at the various stages, from field to laboratory personnel, to detect zoonoses, both new and old.

Information and communication are key components in any prevention and control strategy. Public education and behavioral change are also important factors for successful intervention. Implementing restrictions on anthropogenic animal movement is another important preventive measure. For vector-borne zoonoses, vector control should be an integral part of any intervention strategy.

Interdisciplinary and international collaboration is necessary for the rapid identification and effective management of outbreaks of zoonoses. The pivotal role of international organizations such as World Health Organization and Office International des Epizooties is becoming clearer, exemplified by the 2004 avian influenza outbreak in Southeast Asia. Containing zoonoses with a wildlife reservoir relies on efficient national, regional, and international cross-sectional networks that can improve data sharing and thereby alertness and the timely and effective response to disease outbreaks.
